# Decoding pooled RNAi screens by means of barcode tiling arrays

**DOI:** 10.1186/1471-2164-11-7

**Published:** 2010-01-05

**Authors:** Michael Boettcher, Johannes Fredebohm, Amin Moghaddas Gholami, Yafit Hachmo, Iris Dotan, Dan Canaani, Jörg D Hoheisel

**Affiliations:** 1 Department for Functional Genome Analysis, Deutsches Krebsforschungszentrum, 69120 Heidelberg, Germany; 2 Department of Biochemistry, Tel-Aviv University, 69978 Tel-Aviv, Israel

## Abstract

**Background:**

RNAi screens via pooled short hairpin RNAs (shRNAs) have recently become a powerful tool for the identification of essential genes in mammalian cells. In the past years, several pooled large-scale shRNA screens have identified a variety of genes involved in cancer cell proliferation. All of those studies employed microarray analysis, utilizing either the shRNA's half hairpin sequence or an additional shRNA-associated 60 nt barcode sequence as a molecular tag. Here we describe a novel method to decode pooled RNAi screens, namely barcode tiling array analysis, and demonstrate how this approach can be used to precisely quantify the abundance of individual shRNAs from a pool.

**Results:**

We synthesized DNA microarrays with six overlapping 25 nt long tiling probes complementary to each unique 60 nt molecular barcode sequence associated with every shRNA expression construct. By analyzing dilution series of expression constructs we show how our approach allows quantification of shRNA abundance from a pool and how it clearly outperforms the commonly used analysis via the shRNA's half hairpin sequences. We further demonstrate how barcode tiling arrays can be used to predict anti-proliferative effects of individual shRNAs from pooled negative selection screens. Out of a pool of 305 shRNAs, we identified 28 candidate shRNAs to fully or partially impair the viability of the breast carcinoma cell line MDA-MB-231. Individual validation of a subset of eleven shRNA expression constructs with potential inhibitory, as well as non-inhibitory, effects on the cell line proliferation provides further evidence for the accuracy of the barcode tiling approach.

**Conclusions:**

In summary, we present an improved method for the rapid, quantitative and statistically robust analysis of pooled RNAi screens. Our experimental approach, coupled with commercially available lentiviral vector shRNA libraries, has the potential to greatly facilitate the discovery of putative targets for cancer therapy as well as sensitizers of drug toxicity.

## Background

**Figure 1:**
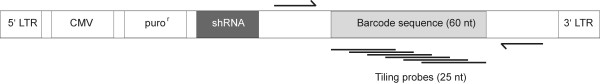
**shRNA expression construct**. Expression of shRNAs from pGIPZ vector constructs is driven by an RNA Polymerase II promoter (CMV). Each shRNA template is associated with a unique 60 nt barcode sequence. Every barcode sequence can be amplified by one primer pair from a pool of constructs. For analysis of pooled RNAi screens, six overlapping tiling probe sequences (25 nt) complementary to each barcode were synthesized on a Geniom One microarray. puro^r^, puromycin resistance gene; shRNA, shRNA template; White areas indicate common sequence among all shRNA expression constructs.

Breast cancer is caused by genetic and epigenetic alterations of the genome, resulting in changes in expression levels of certain genes [1]. In the past two decades, extensive efforts have been undertaken to characterize genes involved in breast cancer development. Genomic alterations and gene expression signatures associated with breast cancer and chemotherapy response have been identified [2-4]. However, genes that are neither mutated nor changed in their levels of expression may also play crucial roles in the progression of breast cancer. One way to identify such essential genes is the inhibition of their expression via RNA interference (RNAi) followed by the analysis of the resulting 'loss-of-function' phenotype. RNAi screens are commonly used to analyze gene function in a variety of model organisms, the most popular ones being *C. elegans *and *Drosophila *[5,6]. More recently, shRNA libraries targeting the human and mouse genome have become available [7,8]. These libraries allow RNAi mediated 'loss-of-function' screens in mammalian cell lines. Pooled RNAi screens have been performed by several groups and revealed a number of cancer cell essential genes [9-11]. The decoding of such pooled RNAi screens by means of microarray analysis has been described previously [12,13]. While some groups employed probe sequences complementary to each shRNAs' specific 21 nt half-hairpin stem sequence [10,11,13] others used unique barcode sequences to analyze pooled shRNA screens [7,9,12]. These 60 nt barcode sequences were cloned adjacent to each shRNA template, allowing the determination of the abundance of individual shRNA templates from a complex pool [7]. Up until now analysis of pooled RNAi screens via barcode sequences was performed by probes complementary to the full length barcode. Here, we introduce the concept of barcode tiling in order to analyze pooled shRNA screens. We synthesized six partially overlapping probe sequences, each 25 nucleotides long, complementary to every unique 60 nucleotide barcode from the pool (Figure 1). This means that the abundance of each shRNA template can be detected from a pool, via hybridization to six different probe sequences rather than just one.

In a series of initial calibration experiments we demonstrate how the barcode tiling approach can quantify the abundance of individual template molecules from a pool of 305 shRNA expression constructs. We further directly compare this new approach of analyzing pooled RNAi screens to the commonly performed analysis via half hairpin probes. We provide evidence that the analysis using barcode tiling probes is not only more sensitive, but also dramatically increases the fraction of analyzable shRNAs from a pool as compared to half hairpin probe analysis.

To further assess the performance of the barcode tiling approach for the detection of essential genes in the breast carcinoma cell line MDA-MB-231, a negative selection screening system was established. We chose to target anti-apoptotic genes which were previously shown to be expressed in either breast carcinoma tissues or normal human breast. From a pooled RNAi screen we identified 28 different shRNA sequences which were depleted from a pool of lentiviral infected cells over a period of four weeks. Finally, eleven potentially inhibitory as well as non-inhibitory shRNAs were selected for individual analysis of their effects on the proliferation of the cell line MDA-MB-231. Validation assays revealed the genes *BIRC5*, *BRCA1*, *HSPA8 *and *NUP62 *to be essential for the viability of the cell line.

The precise profiling of essential genes in cancer cell lines together with their expression pattern, genomic mutations and epigenetic status will lead to a more refined picture of the mechanisms underlying cancer development and the means of eradicating it.

## Results

### Half hairpin versus barcode tiling analysis

In order to assess sensitivity, reproducibility as well as limitations of the barcode tiling approach, we prepared four different template pools with engineered concentrations of individual pGIPZ shRNA expression plasmids. The exact composition of the four templates is summarized in Table 1. In the reference pool a total of 305 expression plasmids were present in equimolar amounts. In the test pools 1 - 3 only 245 constructs constituting subpool-0 remained equimolar, while subpools-1 to -6, consisting of ten constructs each, were diluted by the factors indicated in Table 1. From each of the four pools we separately PCR amplified half hairpin as well as barcode sequences. The resulting PCR product pools were purified, labeled and hybridized to individual DNA microarrays. For both, half hairpin pools as well as barcode pools, exactly the same conditions were used for purification, labeling and hybridization. In order to equalize hybridization properties between barcode tiling probes (25 nt) and half hairpin probes (21 nt), we additionally synthesized 25 nt half hairpin microarray probes, containing 4 nt from the common vector context. We found an approximate 2-fold median array signal intensity increase from 25 nt half hairpin probe sequences as compared to 21 nt probes. Hence we included only 25 nt half hairpin probes into further analysis.

**Table 1:** Scheme of dilution series of pGIPZ shRNA expression plasmids

	Reference pool	Test pool 1	Test pool 2	Test pool 3
Subpool-0	1	1	1	1
Subpool-1	1	9e-01	4e-01	2e-03
Subpool-2	1	1e-03	8e-01	3e-01
Subpool-3	1	1e-04	2e-01	7e-01
Subpool-4	1	1e-01	1e-05	6e-01
Subpool-5	1	5e-01	1e-02	1e-06
Subpool-6	1	4e-03	4e-02	2e-04

Rows show the dilution factors of each of the seven shRNA expression construct subpools in each of the four template pools used for PCR amplification via half hairpin primers or barcode primers respectively. Subpool-0 consists of 245 different pGIPZ plasmids; while subpools-1 to -6 consist of ten different pGIPZ plasmids each.

**Figure 2:**
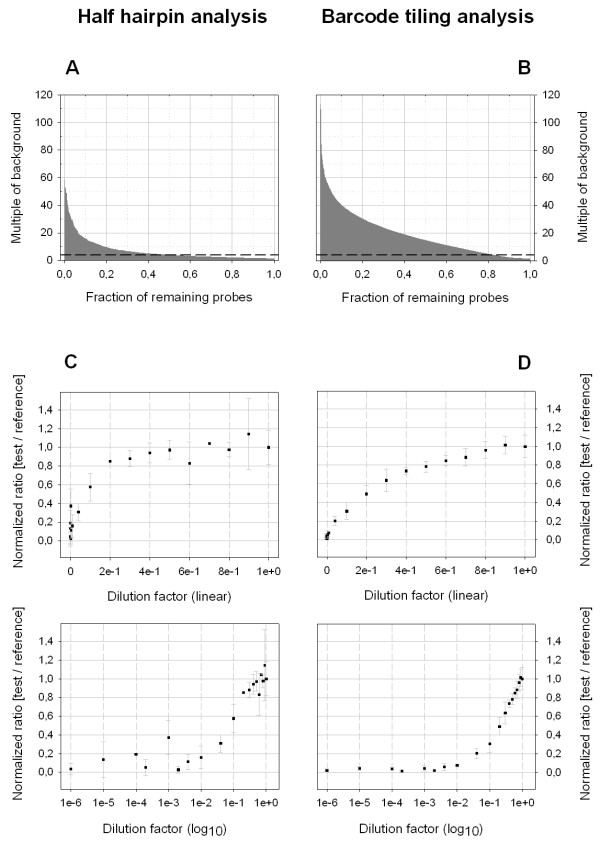
**Comparison between half hairpin and barcode tiling analysis**. Shown are the histograms of probe signal intensities from reference arrays of half hairpin (A) and barcode tiling probes (B) as multiples of the median background intensity. Dashed lines represent a 4-fold median background intensity threshold. Normalized (test/reference) ratios are plotted against the indicated dilution factors in linear (top) and logarithmic scale (bottom) for analysis via half hairpin (C) and barcode tiling probes (D). Values for (test/reference) ratios, standard deviations, *p*-values and number of analyzable constructs per dilution step are shown in Table 2.

Histograms of signal intensities from the hybridized half hairpin as well as the barcode reference pool are shown in Figure 2A and 2B. Absolute signal intensities are displayed as multiples of the reference arrays background signal intensity. We found 49% of the half hairpin probes and 82% of the barcode tiling probes to have signal intensities above a threshold of 4-fold the median background intensity. For further analysis, we included only shRNA expression constructs represented by more than two half hairpin probe replica or more than one barcode tiling probe above the 4-fold threshold in the reference array. Under these conditions we found that 44% of the constructs could be detected via half hairpin probes whereas 92% were detectable by means of barcode tiling probes.

In order to determine the abundance of expression constructs in test pools 1, 2 and 3 we normalized signal intensities from each of the three test arrays to the corresponding signal intensities from the reference array. The calculated (test/reference) signal intensity ratios hence represent a measure for the relative abundance of every shRNA expression construct in each test pool. In a second step, all signal intensity ratios were normalized to the ratios obtained from the equimolar subpool-0 of each array and averaged for every dilution factor. Figures 2C and 2D summarize ratios from all three test pools analyzed via half hairpin or barcode tiling probes respectively. Table 2 further gives an overview of mean ratios together with the corresponding standard deviation, *p*-value and number of analyzable shRNA expression constructs for every dilution step.

**Table 2:** Comparison of half hairpin and barcode tiling analysis


**Half hairpin probes**							**Barcode tiling probes**			
**Dilution factor**	**Mean ratio [test/ref.]**	***σ***	***p*-value**	**n**		**Dilution factor**	**Mean ratio [test/ref.]**	***σ***	***p*-value**	**n**

1e-06	**0.04**	0.06	8e-28	7		1e-06	**0.02**	0.02	4e-80	10
1e-05	**0.14**	0.19	7e-28	6		1e-05	**0.04**	0.02	4e-77	9
1e-04	**0.19**			2		1e-04	**0.04**	0.01	5e-67	9
2e-04	**0.05**	0.09	8e-19	4		2e-04	**0.02**	0.01	3e-90	9
1e-03	**0.37**	0.18	1e-09	4		1e-03	**0.04**	0.03	5e-75	10
2e-03	**0.03**	0.04	5e-23	4		2e-03	**0.02**	0.01	4e-70	8
4e-03	**0.11**	0.08	7e-21	4		4e-03	**0.06**	0.04	7e-80	9
1e-02	**0.16**	0.12	6e-33	7		1e-02	**0.07**	0.01	8e-80	10
4e-02	**0.31**	0.09	2e-17	4		4e-02	**0.20**	0.05	7e-57	9
1e-01	**0.58**	0.15	7e-11	6		1e-01	**0.31**	0.09	9e-40	9
2e-01	**0.85**			2		2e-01	**0.49**	0.09	6e-21	9
3e-01	**0.88**	0.08	3e-01	4		3e-01	**0.64**	0.12	6e-17	10
4e-01	**0.94**	0.10	7e-01	4		4e-01	**0.74**	0.04	1e-09	8
5e-01	**0.97**	0.10	9e-01	7		5e-01	**0.78**	0.06	7e-08	10
6e-01	**0.83**	0.23	6e-02	6		6e-01	**0.85**	0.05	3e-04	9
7e-01	**1.04**			2		7e-01	**0.88**	0.09	9e-03	9
8e-01	**0.97**	0.08	1e+00	4		8e-01	**0.96**	0.09	5e-01	10
9e-01	**1.14**	0.38	9e-02	4		9e-01	**1.01**	0.09	8e-01	8
1e+00	**1.00**	0.18	1e+00	106		1e+00	**1.00**	0.12	1e+00	225

Shown are the normalized (test/reference) mean ratios together with the corresponding standard deviations (*σ*), *p*-values and numbers of analyzable shRNA expression constructs (n) for each dilution factor. The *p*-values reflect significance of the differences between mean ratios obtained from the undiluted subpool-0 and the indicated dilution factors. For n < 3, no standard deviation or *p*-value could be determined.

### Negative selection screen

In order to assess the performance of barcode tiling arrays in negative selection screens, we established a screening system to detect essential genes in the breast carcinoma cell line MDA-MB-231. For that purpose, lentiviruses carrying each of the 305 different shRNA expression constructs, targeting 121 individual antiapoptotic genes (see Additional file 1), were pooled. This lentiviral mix was used to infect MDA-MB-231 breast carcinoma cells at a low multiplicity of infection (MOI) of 0.3, while selecting for puromycin. The low MOI ensured most cells would carry a maximum of one knock-down construct targeting a single gene. After five days of puromycin selection, total high molecular weight (HMW) DNA was extracted and served as a reference pool (t_zero_). Another cell fraction was cultured for an additional four weeks and then subjected to HMW DNA extraction, representing the test pool (t_end_). The barcode sequences from t_zero _and t_end _of the pooled screen were recovered by means of PCR on HMW genomic DNA, labeled and hybridized to two individual barcode tiling arrays. To account for differences in viral titers as well as in PCR amplification and hybridization efficiencies of individual probe sequences, all probe signal intensities from the test pool (t_end_) were normalized to the reference pool from time point zero (t_zero_) by calculating the (t_end_/t_zero_) ratio. Lower titers of individual viruses in the viral pool for example would result in lower t_zero _as well as t_end _signal intensities. If the titer of a particular virus at time point zero was too low, the corresponding t_zero _tiling probe signals would not exceed the selected threshold and hence these shRNA expression constructs would be excluded from further analysis. By applying a threshold of 10-fold the median background intensity for probe signals in the t_zero _reference array we avoided the described problems. Additionally, a high threshold is important for the t_zero _reference pool in order to provide the dynamic range necessary to quantify the abundance of shRNA expression constructs in the test pool t_end_.

### Correspondence analysis of the negative selection screens

**Figure 3:**
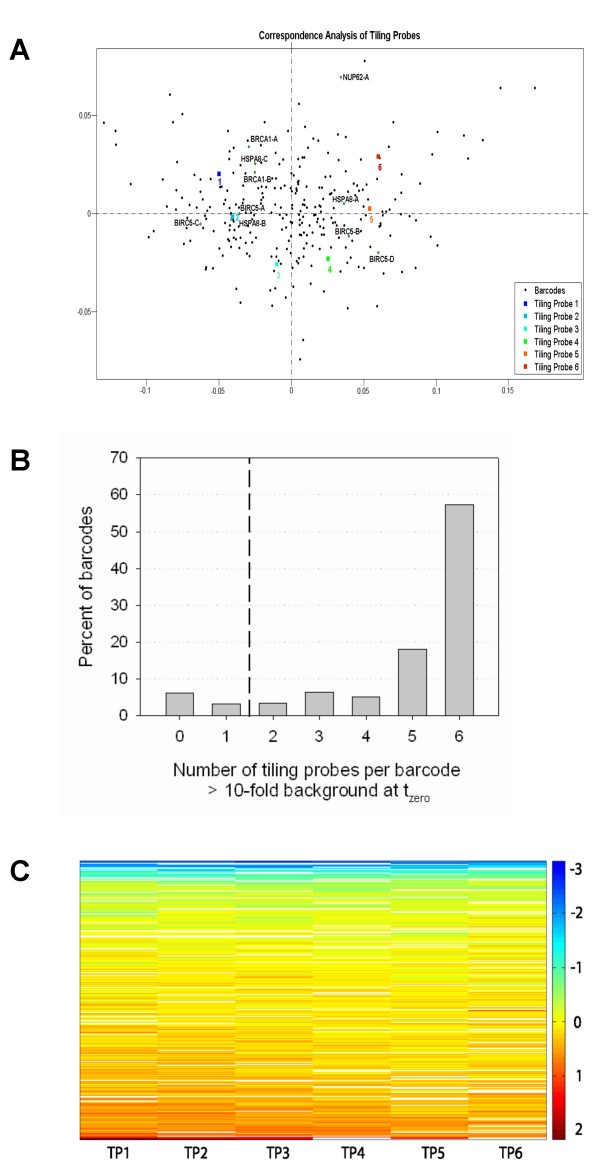
**Barcode tiling probe performance in negative selection RNAi screens**. A - Colored squares represent the six tiling probes complementary to each barcode sequence. Black spots represent signal intensity profiles at time point zero from each of the barcode sequences included in the pooled screen. The intersection of the dashed lines marks the position of the centroid. Validated candidate constructs are highlighted. B - Tiling probes with reference (t_zero_) signal intensities below a 10-fold median background intensity threshold were excluded from analysis. Shown are percentages of 305 shRNA expression constructs included in the pooled screen that were detectable by the indicated number of barcode tiling probe sequences above the 10-fold threshold. For 91 percent of the shRNA expression constructs more than one barcode tiling probe matched those criteria (right of the dashed line). Only those barcodes were considered for further analysis. Percent values represent absolute counts from Additional file 2 relative to the total number of 305 shRNA expression constructs. C - A heat map was generated from log_2 _ratios obtained from each tiling probe (TP) that passed the filter criteria, corresponding to log_2 _values in Additional file 2. Columns represent the six different tiling probes and lines the 278 barcode sequences retained after filtering sorted by their tiling probe mean log_2 _values. White cells represent tiling probes which did not pass the described filter criteria.

Associations between tiling probes and barcode sequences were analyzed by means of correspondence analysis. Correspondence analysis aims to separate dissimilar objects, in our case tiling probe sequences as well as barcode sequences, from one another [14]. Thus, similar objects are clustered together resulting in small distances, whereas dissimilar objects are located further apart. A projection of this analysis is shown in Figure 3A where time point zero signal intensities from all 305 barcodes were used to determine the association between each of the six different tiling probes representing every barcode, marked as colored squares. Expectedly, contiguous tiling probes, sharing the highest similarity with one another, are located closer to each other than tiling probes sharing no sequence similarity.

In a second step, all barcodes, represented as black dots, were spaced in the projection according to their association with each of their six tiling probes. Strongest signal intensity from one particular tiling probe as compared to the remaining five means strongest association of the barcode with this tiling probe. For positive associations of a barcode with a particular tiling probe, both objects are located in the same direction from the centroid. The larger the distance from the centroid, the stronger the associations between the barcode and the given tiling probe. For negative associations, each of the two objects lies on opposite sides of the centroid. This means, barcodes are spaced in the projection according to their signal intensity profiles at time point zero. An example of strong association is given by the barcode sequences from constructs BIRC5-A and HSPA8-B, highlighted in the projection. Both barcodes show a positive association with tiling probe two and, at the same time, a negative association with tiling probes four, five and six. In other words, t_zero _signal intensities detected from tiling probe two were much stronger for both barcodes than signal intensities detected from tiling probes four, five and six. Interestingly, no general preference for any of the tiling probes was detected, as represented by the equal distribution of all vector profiles in the projection.

### Identification of candidate essential genes

The depletion of a certain barcode over the time of the screen is expected to result in a decreased (t_end_/t_zero_) ratio and thus indicate that the associated shRNA targeted a gene which was essential for the proliferation of the cell line MDA-MB-231. Therefore, log_2 _signal intensity ratios (t_end_/t_zero_) were calculated from all signals that passed the described t_zero _filter criteria and averaged for each tiling probe sequence individually. In total, three independent replicate microarray experiments were carried out, resulting in a maximum of nine signal intensity ratios for each tiling probe. Tiling probes represented by less than four out of the possible nine replicate signal ratios were discarded. A summary of all determined log_2 _ratios is shown in Additional file 2 and raw microarray data is accessible through ArrayExpress (Additional file 3). Figure 3B further gives an overview of the fractions of barcodes that were detectable by the indicated number of tiling probes. Expression constructs represented by at least two barcode tiling probes were considered for further analysis. Altogether, out of 305 shRNA expression constructs included in the pool, 278 (91%) could be analyzed by means of the described criteria.

**Figure 4:**
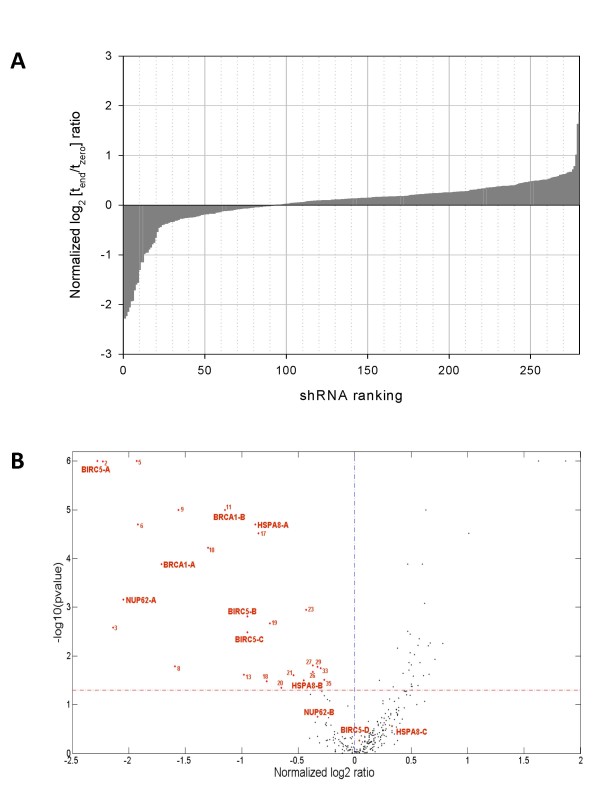
**Overview of results from negative selection screens**. A - The log_2 _(t_end_/t_zero_) signal intensity ratios were calculated from all probes that passed the described filter criteria and averaged for each shRNA expression construct. Negative log_2 _ratios indicate the depletion of cells expressing a particular shRNA from the pool of cells, following the four weeks of the screen. B - The log_2 _ratios determined for each shRNA expression construct were plotted against their significance. The red dashed line represents a *p*-value of 0.05 which was used as cut off. Highlighted in red are significant candidate shRNAs (numbers) and validated candidate constructs. The indicated numbers correspond to the numbers given in the Additional file 2.

A heat map of all log_2 _ratios from Additional file 2 is shown in Figure 3C. Lines represent the 278 shRNAs sorted by the mean value of their corresponding log_2 _ratios from tiling probes retained after filtering. Table 3 further shows the correlation coefficients (*r²*) between different tiling probes. As expected, correlation between log_2 _ratios from contiguous tiling probes is highest (*r² *= 0.84, +/-0.02; Table 4) since they share the highest sequence similarity. With a decrease in sequence similarity, correlation also decreases. Thus, tiling probes sharing no common sequence display the lowest correlation (*r² *= 0.68, +/-0.02). A ranking of the mean log_2 _ratios, representing the abundance of each shRNA in the pool after four weeks of screening, is shown in Figure 4A. Those log_2 _ratios were then plotted against their significance. The volcano plot in Figure 4B gives an overview of the results from our pooled screen. It shows the distribution of log_2 _ratios determined for each shRNA, relative to their calculated *p*-values. We found that 28 candidate constructs showed negative log_2 _ratios together with a *p*-value < 0.05, indicating their depletion from the pool.

**Table 3:** Cross-correlation between tiling probes.

TP1	TP2	TP3	TP4	TP5	TP6	Tiling probe
	0.86	0.70	0.68	0.67	0.67	**TP1**
		0.84	0.78	0.72	0.70	**TP2**
			0.86	0.74	0.68	**TP3**
				0.84	0.75	**TP4**
					0.81	**TP5**
						**TP6**

Correlation coefficients (*r²*) between log_2 _ratios from all analyzed tiling probes (TP) are shown.

**Table 4:** Impact of probe sequence similarity on correlation.

Sequence similarity (nt)	*r^2 ^*mean	*σ*
0	0.68	0.02
4	0.69	0.02
11	0.74	0.03
18	0.84	0.02

The mean *r² *values from all tiling probes sharing 18, 11, 4 nt or no sequence similarity is shown. Correlation is strongest between probes with a sequence overlap of 18 nt and decreases with reduced sequence similarity.

### Validation of candidate essential genes

**Figure 5:**
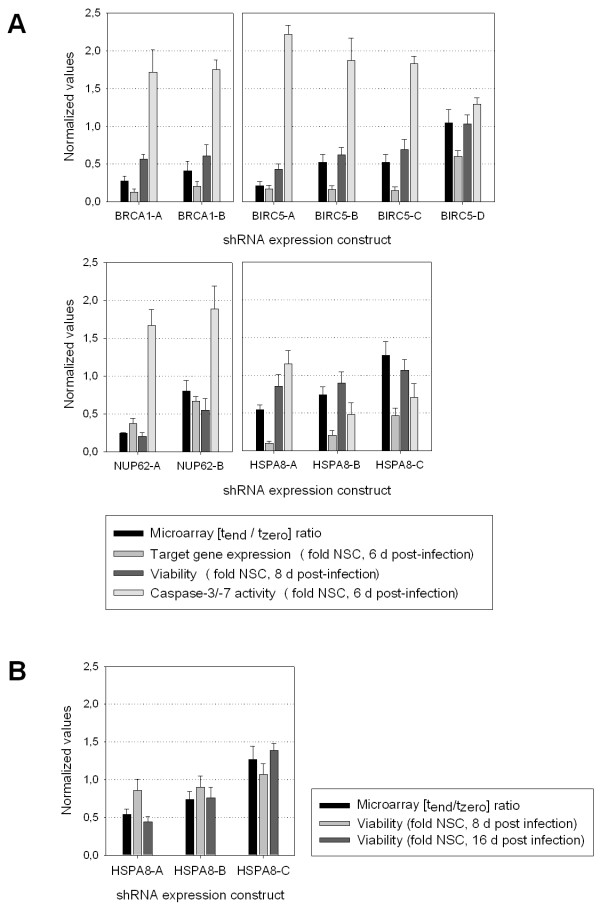
**Validation assays of individual candidate constructs**. A - Shown are the (t_end_/t_zero_) ratios from the pooled screen, the target gene expression relative to the NSC at six days post-infection, the cell viability relative to the NSC at eight days post-infection, and the activity of caspase 3/7 relative to NSC as a measure of apoptosis induction at six days post infection. NSC, non silencing control. B - Shown are the (t_end_/t_zero_) ratios determined over four weeks of the pooled screen, together with the cell viability relative to the NSC at eight and sixteen days post-infection. The inhibition of *HSPA8 *leads to a more pronounced decrease in viability at sixteen days as compared to eight days post infection, in accordance with the (t_end_/t_zero_) ratios. NSC, non silencing control.

To verify the potential anti-proliferative effects of candidates identified through the analysis of the pooled RNAi screen, we selected eleven shRNA expression constructs for closer analysis in an arrayed 96-well format. First of all, two shRNA expression constructs, termed BRCA1-A and BRCA1-B, both encoding identical shRNA sequences targeting the expression of *BRCA1*, but associated with two different 60 nt barcode sequences were selected for validation. The log_2 _ratios from both constructs indicated a significant anti-proliferative effect [(BRCA1-A (-1.706, *p *= 1.3e-4)/BRCA1-B (-1.145, *p *= 1e-5)]. We transduced the constructs individually into the host cell line and examined their potential to reduce target mRNA abundance, inhibit cell viability and induce apoptosis. For BRCA1-A as well as for BRCA1-B we detected close to equal reduction of *BRCA1 *expression, a concomitant decrease in cell viability and induction of caspases 3/7, a hallmark of apoptosis (Figure 5A).

In much the same way as for *BRCA1*, further constructs targeting expression of the genes *BIRC5 *(BIRC5-A-D), *NUP62 *(NUP62-A-B) and *HSPA8 *(HSPA8-A-C) were analyzed. For each of the three genes we identified at least one construct with a significant log_2 _ratio below -0.5 and one construct showing a ratio greater than -0.5. Expression levels were reduced below 0.4-fold that of the non-silencing control (NSC) by at least one construct targeting each of the three mentioned genes. Cells with efficient reduction of *BIRC5 *and *NUP62 *expression were strongly impaired in their viability when assayed eight days post-infection (BIRC5-A-C/NUP62-A-B). In the case of *HSPA8*, a reduction of mRNA expression to 0.1-fold that of the NSC caused only a mild reduction in cell viability (HSPA8-A). Moreover, transduction of BIRC5-A-C as well as NUP62-A-B induced caspases 3/7 unlike any of the *HSPA8 *targeting constructs (Figure 5A).

The weak inhibition of viability after reduction of *HSPA8 *expression was unexpected considering the log_2 _ratios from HSPA8-A (-0.885, *p *= 2.0e-5) and HSPA8-B (-0.446, *p *= 3.2e-2). A major discrepancy between both assays, however, is their duration. While the pooled screens were carried out over a period of four weeks, validation assays were performed eight days post infection. To test for the possibility of *HSPA8 *knock-down impairing viability later than eight days post infection, we decided to perform another viability assay for the constructs HSPA8-A-C at sixteen days post infection. As shown in Figure 5B, inhibition of viability was detected for HSPA8-A as well as for HSPA8-B, resembling the (t_end_/t_zero_) ratios determined from the pooled screen.

## Discussion

### Technical considerations

In this manuscript we demonstrate how our barcode tiling approach facilitates highly reproducible and quantitative analysis of pooled RNAi screens. As compared to previous approaches employing a single half-hairpin or full length barcode probe [9-11,15,16], we used six non-identical tiling probe sequences to measure the abundance of shRNA expression constructs from three test pools of pGIPZ plasmids with engineered concentrations. We directly compared our approach to the analysis of the same pools via half hairpin probe sequences. When we apply a threshold of 4-fold the median background intensity to the reference array from the half hairpin as well as the barcode tiling probes, we retain 49% of the half hairpin probes and as many as 82% of the barcode tiling probes. These values are similar to the findings from Silva et al. who determined 60% of the half hairpin and 80% of the full barcode sequences to exceed 4-fold background intensity [9]. One possible reason for the reduced fraction of half hairpin as compared to barcode reference signals passing the threshold is the PCR reaction used to amplify the molecular tags. Due to the complementary nature of shRNA sequences, self-annealing could be an explanation for the reduced signal intensity. Seeing as the forward primer binding site in the loop sequence of the shRNA consists of only 19 nucleotides, a rather low annealing temperature of 50°C had to be used. The stem of the shRNA, however, is 21 nt long. Hence, a sequence dependent, selective self-annealing of specific shRNAs could result in inefficient PCR amplification of those half hairpin sequences from a pool. As a consequence, the probe signal in the reference pool will decrease below the 4-fold threshold and be excluded from further analysis. Given that no such self-complementary sequences are found in the barcodes, a more equal amplification of individual sequences from a pool is likely.

Further advantages arise from the size of the 60 nucleotide long barcode sequence. Tiling the sequence into six 25 nt long probes allows the omission of regions in the barcode with unfavorable hybridization properties. Seeing as six dissimilar tiling probes represent each barcode, identical signal intensities from all six tiling probes would be expected, if hybridization properties between them were equal. As illustrated by the distribution of vector profiles in Figure 3A, signal intensities obtained from the six different tiling probes representing every barcode vary dramatically, indicating very different hybridization properties of different tiling probes. Applying a high 10-fold background threshold to the t_zero _reference pool excluded tiling probes with weak signal intensities from further analysis. As summarized in Figure 3B, for 57% of the cases, all six barcode tiling probes passed the 10-fold background threshold. For another 34%, however, at least one tiling probe did not exceed the threshold, resulting in only two to five analyzable tiling probes per barcode. In total we could analyze 91% of shRNA expression constructs using more than one tiling probe with a t_zero _signal above the 10-fold threshold from the negative selection screens. Similarly, when applying a 4-fold background threshold to the pGIPZ plasmid reference pool, we detected 92% of the shRNA expression constructs with more than one tiling probe. This is a substantial increase compared to the 44% of shRNA expression constructs analyzable via half hairpin probe sequences.

Besides an enhanced fraction of analyzable shRNA expression constructs, the detection by means of barcode tiling probes also increases the statistical robustness of the analysis. Seeing as the abundance of each shRNA expression construct is detected by at least two, in most cases even six different tiling probes, variations resulting from probe sequence biases are minimized. This is reflected by the lower standard deviations as well as *p*-values when comparing barcode tiling with half hairpin probe results (Table 2). Additionally, the correlation coefficients presented in Table 4 clearly point out the difference in log_2 _ratios obtained from different tiling probes of the same barcode. If probe sequence properties had no impact on the determined log_2 _ratios, the correlation coefficient should not decrease with decreased sequence similarity. However, we found correlation between log_2 _ratios from probes sharing 18 out of 25 bp nucleotide sequence similarity to be *r² *= 0.84. The correlation between tiling probe sequences decreased further with reduced similarity (Table 4). When detecting the abundance of shRNA expression constructs via half hairpin probes on the other hand, each (test/reference) ratio is determined based on one single probe sequence. Consequently, the variance of mean values determined from the pGIPZ plasmid dilution series is generally greater, when analyzing the pools via half hairpin as compared to barcode tiling probes (Table 2). Incorporating signals from different tiling probes reduces sequence biases and allows more accurate detection of the abundance of individual shRNA expression constructs from a pool. Figure 2D further illustrates how barcode tiling analysis yields highly reproducible (test/reference) ratios that allow quantification of the relative abundance of individual shRNA expression constructs over a large data area, ranging from 7e-1 to 1e-2. The first test dilution factor that can be distinguished from the undiluted reference with high significance (*p *< 1e-2) is 7e-1 (Table 2). Any test concentration below 1e-2 fold the reference concentration resulted in a (test/reference) ratio below 0.07. This goes to show that barcode tiling analysis can not only quantify shRNA expression construct abundance over a large data area, but also strongly reduces chances to detect false positives as well as false negatives. In comparison, half hairpin analysis allows quantification, if at all, only in a more limited data area (1e-1 to 1e-2), together with decreased reproducibility, making false positive as well as negative detection more likely (Figure 2C).

In summary, comparing half hairpin with barcode tiling probe analysis of the same templates highlights the differences between both analysis methods. A dramatic increase in the fraction of analyzable constructs together with much more statistically robust and accurate (test/reference) ratios clearly demonstrates the advantages of the barcode tiling approach over the customary half hairpin analysis.

### Negative selection screen

For a negative selection screen, the 305 pGIPZ plasmids were packaged into lentiviral particles and a pool of virus was used to infect the breast carcinoma cell line MDA-MB-231. In an initial calibration step, we discarded probe sequences displaying t_zero _signal intensities that were below 10-fold background, as compared to a 4-fold background used for the analysis of the engineered pGIPZ plasmid pools. This resulted in only nine percent of the shRNA constructs from the pooled screen which did not fulfill the criteria for further analysis (Figure 3B). Similarly, analysis of the equimolar pGIPZ reference pool, which contained all 305 expression constructs, resulted in eight percent of the shRNA expression constructs not fulfilling the described criteria. These finding indicate that we either had incorrect barcode sequence information (partly obtained from Open Biosystems Inc.), resulting in non-complementary probe sequences on the microarray, or problems with PCR amplification of the barcodes represented by the undetectable probe signals and that low titers of individual viruses in the pool were not responsible for undetectable shRNA expression constructs.

From 28 candidate shRNAs identified from the pooled negative selection screen to potentially inhibit the viability of the breast carcinoma cell line MDA-MB-231, a subset was selected for arrayed validation assays. We found that reduced *BRCA1 *expression resulted in caspase 3/7 induction and decreased viability of the cells. These findings are in accord with the essential role of *BRCA1 *in embryonic cell proliferation [17]. Paradoxically, under non-physiological over-expression conditions, *BRCA1 *induces apoptosis, and its silencing increases viability of certain cancer cells [18,19]. Our findings indicate that in MDA-MB-231, *BRCA1 *inhibition might be more detrimental than in other cell lines. In this context it is also worth mentioning the potential role of the *BRCA1 *binding partner *BARD1 *as an essential gene for MDA-MB-231 cell growth. From our DNA microarray data analysis we found the log_2 _ratio for the *BARD1 *targeting construct V2LHS_93186 to be as low as -2.228 (*p *= 1.01e-6). Interestingly, *BARD1 *has been described before as being essential for the function of *BRCA1 *and the survival of embryonic mice [20]. Taken together, our data suggests an important role for functional *BRCA1 *pathways in MDA-MB-231 cell viability. Besides the tumor suppressor *BRCA1*, we identified three more candidate genes whose expression was demonstrated to be of importance for the proliferation of the breast cancer cell line MDA-MB-231. Among those genes was the inhibitor of apoptosis *BIRC5*, the nuclear pore complex (NPC) component *NUP62 *and the heat shock protein 70 family member *HSPA8*.

*BIRC5 *is known to be an Inhibitor of Apoptosis (IAP) that is over expressed in numerous human cancers including breast cancer [21]. It has been claimed that *BIRC5 *has the potential to be a prognostic marker in breast cancer patients [22]. Furthermore, the inhibitory effects on the proliferation of MDA-MB-231 after siRNA mediated silencing of *BIRC5 *have been documented [23]. Here we confirm that inhibition of *BIRC5 *expression by shRNA below 0.2-fold of its endogenous level strongly inhibits proliferation of MDA-MB-231 cells via caspase 3/7 activation. These findings provide further evidence for the potentially essential role of *BIRC5 *in human breast cancer.

The ubiquitously expressed *NUP62 *has been described to be an essential part of the Nuclear Pore Complex. It has been reported to be involved in cargo transport across the nuclear envelope [24]. Importantly, recently a role for *NUP62 *in cell cycle regulation has been proposed [25]. Here we demonstrate that *NUP62 *knock-down leads to induction of apoptosis, together with a decrease in viability.

**Figure 6:**
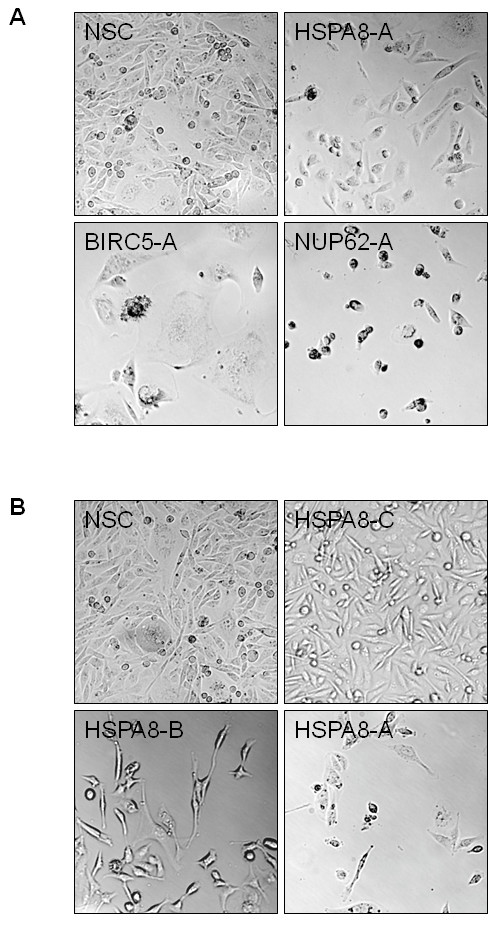
**Microscopic images of altered cell morphology after efficient knock-down of the indicated genes**. A - Cells at eight days post infection with the indicated shRNA expression constructs. While in the case of NUP62-A we observed the typical apoptosis phenotype of MDA-MB-231, in the cases of BIRC5-A and HSPA8-A the morphology was very different. *BIRC5*-knock-down cells were several times larger than cells expressing the NSC, and *HSPA8 *knock-down cells displayed a fibroblast-like phenotype. NSC, non silencing control. Magnification is 10x. B - Sixteen days post infection cells expressing HSPA8-A were strongly impaired in their viability (see Figure 5B).

Finally, we identified the heat shock cognate protein *HSPA8 *(Hsc70) to be important for the viability of MDA-MB-231 cells. The highly conserved protein can bind to nascent polypeptides and facilitate their correct folding. It is ubiquitously expressed in the cytosol of a variety of non-tumor as well as cancerous cells including breast cancer [26]. It has also been described by Rohde et al. (2005) that the knock-down of *HSPA8 *in HeLa cells generated an elongated fibroblast-like morphology before rounding up and detachment from the culture dish. In concordance with those findings we observed a very similar phenotype in MDA-MB-231 after efficient *HSPA8 *knock-down at eight days post-infection (Figure 6A). However, viability was only slightly impaired at that time point. Thus we decided to record another time point at sixteen days post-infection. Indeed, we could show a much more pronounced inhibition of viability at sixteen days post infection with HSPA8-A and HSPA8-B (Figure 5B), as predicted from their (t_end_/t_zero_) signal intensity ratio. Additionally, MDA-MB-231 cells infected with HSPA8-A also detached from the cell culture dish at sixteen days post infection, which is again consistent with the findings from Rohde et al. (Figure 6B).

**Figure 7:**
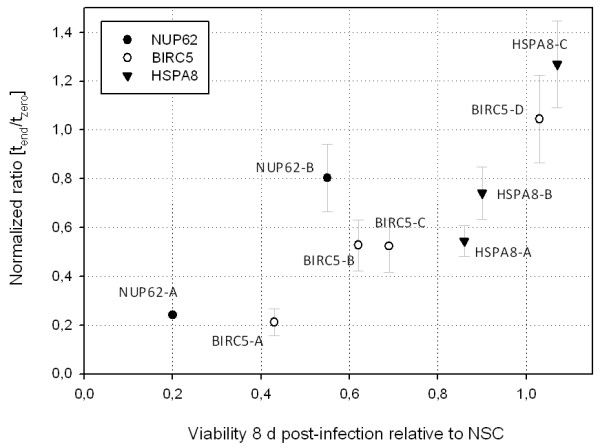
**Results from pooled screens compared to viability data from selected shRNA expression constructs**. Shown is the correlation between (t_end_/t_zero_) signal intensity ratios from pooled screens and cell viability assays. While the (t_end_/t_zero_) ratios represent inhibition of proliferation over a period of four weeks, the viability values only cover eight days. Thus this plot displays differences in the immediacy by which the knock-down of different genes leads to inhibition of proliferation. Constructs are the same as in Figure 5A.

Taken together, our data illustrates how inhibiting the expression of different essential genes can influence the proliferation of MDA-MB-231 at different immediacy. While, for example, the log_2 _ratios determined via microarray analysis of pooled screens from the constructs BIRC5-B (-0.948, *p *= 1.5e-3), BIRC5-C (-0.954, *p *= 3.2e-3) and HSPA8-A (-0.885, *p *= 2.0e-5) are almost identical, their viability at eight days post infection varies greatly (Figure 5A). Both constructs targeting *BIRC5 *show similar reduction of cell viability at eight days post infection (BIRC5-B = 0.62 +/-0.1 and BIRC5-C = 0.69 +/- 0.14), whereas the construct HSPA8-A shows much weaker effects (0.86 +/-0.15). The inhibitory effect of HSPA8-A only becomes noticeable at sixteen days post infection (Figure 5B). To further illustrate this issue, we plotted the viability data against the (t_end_/t_zero_) ratios for the constructs we used for validation assays (Figure 7). While the ratio determined from the pooled screen represents a measure for proliferation over 33 days, the viability assay only detects effects that occur within eight days post-infection. If knock-down of *HSPA8*, *BIRC5 *and *NUP62 *induced inhibition of proliferation with the same immediacy one would expect all data points to be on one linear regression line. However, whereas depletion of some genes takes no longer than eight days to almost completely inhibit the viability of the infected cells (e.g. NUP62-A), some others take a longer period of time (e.g. BIRC5-A, HSPA8-A). These differences are further reflected by the striking morphological changes in cells eight days post infection with the constructs NUP62-A, BIRC5-A and HSPA8-A (Figure 6A). Introduction of NUP62-A, for instance, resembles the typical apoptosis phenotype of MDA-MB-231 leading to small round cells that finally detach from the cell culture dish. Introducing BIRC5-A, on the other hand, results in cells much larger than the control cells which are unable to divide but do not detach from the surface until eight days post infection. HSPA8-A cells, finally, seem to be only slightly impaired in their ability to divide but display the fibroblast-like morphology described for HeLa cells by Rohde et al (2005) before detaching from the surface at sixteen days post infection (Figure 6B).

## Conclusions

In the work presented, we demonstrate how pooled RNAi screens can be quantitatively and reproducibly analyzed by means of barcode tiling arrays. We clearly show the advantages of this novel method over the commonly performed analysis via half hairpin arrays. To further exploit the full potential of barcode tiling, optimal tiling probe sequences need to be experimentally determined for each barcode present in a given shRNA expression library. This calibration step would ensure a maximized fraction of analyzable expression constructs combined with a reduced sequence bias as compared to currently used approaches.

Besides essential gene discovery, a variety of additional exciting applications for pooled RNAi screens become conceivable with the help of increased sensitivity obtained from barcode tiling analysis. One intriguing idea, for example, are pooled synthetic lethality screens [27,28] allowing the identification of cancer specific molecular targets. Such screens require accurate methods for the detection of particular shRNA abundance. Our work provides the methodological scaffolding to allow the analysis of such technically challenging experiments.

## Methods

### Lentiviral pool production and pooled negative selection screen

HEK 293T cells were seeded in 96 well microplates at 2 × 10^4 ^cells per well and co-transfected with 100 ng of each of 305 individual pGIPZ human shRNA encoding lentiviral plasmids (Open Biosystems), 50 ng psPAX2 and 25 ng pMD2.G plasmids (kindly provided by Prof. Trono), respectively. Viruses were harvested 48 h and 72 h post-transfection, pooled and stored at -80°C. The viral titer of the pool was determined to be 6 × 10^4 ^units/ml. MDA-MB-231 cells were seeded in triplicate at 7 × 10^5 ^cells per 150 cm² cell culture flasks in standard cell culture medium (DMEM, 10% FCS, 1% penicillin/streptomycin 10,000 U). Twenty four hours post seeding 30 ml culture medium containing 8 μg/ml polybrene was added to each of the triplicates mixed with 3.3 ml of the viral pool to achieve a multiplicity of infection (MOI) of 0.3. Twenty four hours post-infection the viral supernatant was aspirated and replaced with culture medium containing 0.5 μg/ml puromycin. Seventy two hours post puromycin selection infected cells were seeded into 150 cm² flasks at 3.5 × 10^5 ^cells per flask. The remaining cells were harvested from each of the three biological replicates and stored in aliquots of 1.5 × 10^6 ^cells per replicate at -80°C for HMW DNA purification (t_zero_). The selected cells were cultured in 0.5 μg/ml puromycin medium for 28 additional days after t_zero_. 2 × 10^5 ^cells were transferred into fresh 150 cm² cell culture flasks when 80% confluent, representing approximately 600 copies of each barcode in each triplicate. From every passage, pellets of 1.5 × 10^6 ^cells were harvested and stored at -80°C.

### Barcode and half hairpin DNA amplification and labeling

From each of the triplicate cell pellets harvested at five days (t_zero_) and 33 days (t_end_) post-infection, HMW DNA was extracted using the QIAamp DNA Micro Kit (Qiagen) according to the manufacturer's instructions. On average total amounts of HMW DNA extracted from 1.5 × 10^6 ^cells were around 3 μg from each of the three biological replicates. The purified DNA was eluted in AE buffer and adjusted to 50 ng/μl. Each unique 60 nucleotide barcode DNA sequence was PCR amplified from 100 ng genomic DNA template for each biological triplicate. Assuming a weight of 3 pg per genome, this represents an average of 350 copies per barcode. Seeing as microarray experiments were performed in independent triplicates, including PCR amplification of the barcode sequences, each barcode was represented by an average of 1,050 copies in total. Barcode sequences were amplified via PCR reactions using 0.4 μM 5' primer BC-For [5'- AACTGAATACCTTGCTATCTCTTTGA-3'] and 0.4 μM 3' primer BC-Rev [5'-TCCAGAGGTTGATTGTTCCA-3'], 250 μM of each dNTP (Fermentas), 1x HotStart Buffer (Qiagen), 1x Q-Solution (Qiagen), 1.5 mM MgCl_2_, 2.5 units HotStart polymerase (Qiagen) and in a total volume of 100 μl. Thermal cycler PCR conditions were 95°C for 15 min followed by 42 cycles of 95°C for 40 sec., 58°C for 2:00 min., 72°C for 1:30 min. and finally 72°C for 10 min. PCR amplification from pGIPZ plasmid pool templates was essentially performed in the same way as from genomic DNA, only that the copy number was adjusted to 1,500 copies per equimolar pGIPZ construct (6 pg/100 μl PCR). For half hairpin amplification we used the 5' primer HH-For [5'-TAGTGAAGCCACAGATGTA-3'] and the 3' primer HH-Rev [5'-CTAAAGTAGCCCCTTGAATTC-3']. In a gradient PCR we determined the optimal annealing temperature for the half hairpin primer pair to be 50°C. PCR products were purified using QIAquick PCR Purification Kit (Qiagen) eluted in H_2_O and adjusted to 50 ng/μl. 150 ng of the PCR product from each triplicate were pooled and incubated together with 30 ng/μl random primer oligonucleotides (Invitrogen) in a total volume of 28 μl at 99°C for 5 min. After the denaturation step 1x reaction buffer (1 M Hepes pH 6.6, 250 mM Tris-HCl pH 8.0, 25 mM MgCl_2_, 50 mM 2-mercaptoethanol), 2 mM of each dATP, dCTP, dGTP and 1.3 mM dTTP, (Fermentas) together with 0.7 mM biotinylated-dUTP (Roche), 0.4 mg/ml BSA (Sigma) and 7.5 units Klenow fragment (New England Biolabs) was added to a total volume of 40 μl. After incubation at 37°C for 3 h and 75°C for 10 min, 4 μl of 3 M sodium acetate (pH 5.6) and 100 μl ethanol were added and the DNA was precipitated at -80°C for 2 h. After centrifugation at 18,320 × g for 20 min the supernatant was aspirated, the pellet was dried and resuspended in 15 μl 1x hybridization mix (100 mM 2-[N-morpholino]ethanesulfonic acid (MES), 0.9 M NaCl, 20 mM Na_2_EDTA, 0.01% (v/v) Tween-20, 0.5% BSA 0.1 mg/ml herring sperm DNA (Febit).

### Microarray design and hybridization

We used the photo-controlled *in-situ *synthesis technology Geniom One (Febit Biomed GmbH) for synthesis, hybridization and detection of microarrays [29]. The Geniom One microarray is divided into eight individually accessible subarrays allowing the analysis of eight samples in parallel. Half hairpin probes were synthesized in quadruplicates as 21 nt sequence as well as 25 nt sequences containing additional 4 nt from the common mir-30 sequence at their 3' end. As for the barcode sequences, probes the length of 25 nt were synthesized complementary to each 60 nt barcode. Every barcode was covered by six probes in seven nucleotide jumps. Three replicates of each probe were synthesized in each subarray, resulting in 18 probes representing one barcode. In total 5490 probes were synthesized to detect barcodes associated with 305 different shRNA expressing constructs. Additionally eleven half hairpin and 66 tiling probes that did not match any barcode sequence were synthesized in triplicates as negative controls. Before hybridization the biotinylated barcode fragments in 1x hybridization mix were heated to 95°C for 3 min then placed on ice for 1 min. The denatured targets from t_zero _and t_end _were then applied to individual subarrays of the Geniom One microarray and incubated at 45°C for 16 h. After washing routines according to the Febit protocol, each subarray was incubated with 5 μg/ml streptavidin phycoerythrin (Invitrogen) in 6x SSPE (0.9 M NaCl, 60 mM NaH_2_PO_4_, pH 7.4 and 6 mM Na_2_EDTA). Signal intensity detection was performed using the inbuilt CCD camera of the system and local backgrounds were subtracted by means of internal Geniom One software routines.

### Data analysis

Median background signal intensities were determined from half hairpin or barcode tiling probe sequences complementary to eleven shRNA expression constructs that were absent in the analyzed pools. Thresholds from multiples of those background intensities were applied as described. Signal intensities from each probe after local background subtraction were normalized to the median signal intensity of each subarray. The mean signal intensity ratios for each half hairpin or tiling probe were calculated from the remaining probes by dividing the signals from probes of the test-subarray by their corresponding reference-subarray probe signals. Finally, the mean ratio from all tiling probes representing one barcode was determined. The analysis of the negative selection screen was performed in three independent replicates. Their mean values were calculated as a measure of relative barcode abundance and hence the anti-proliferative effect of associated shRNAs. Candidates with biologically significant signals were identified using linear models in the limma package [30] for the significance analysis of microarray data. Coefficients, moderated t-statistics and corresponding *p*-values for testing all possible contrasts were calculated using Empirical Bayesian methods. We used appropriate design matrixes for the linear model fitting. We then performed pair-wise comparisons between time point zero and time point end tiling probes by means of contrast matrix. The *p*-values for the coefficients of interest were adjusted for multiple testing by means of Benjamini and Hochberg's algorithm [31], which controls the expected false discovery rate (FDR) below the specified value.

### Quantitative RT-PCR

MDA-MB-231 cells were seeded in six-well microplates at 10^4 ^cells per well. After 24 h, 90 μl of lentiviral supernatant (approx. 6000 units) in culture medium containing 8 μg/ml polybrene was added to the cells to achieve a MOI < 1. Twenty four hours later the lentiviral medium was aspired and replaced by culture medium containing 0.5 μg/ml puromycin. At day six post-infection, cell pellets were collected and total RNA was isolated using the RNeasy Mini Kit (Qiagen). One microgram of total RNA from each sample was used for first strand cDNA synthesis by Superscript III (Invitrogen). The QuantiTect SYBR Green PCR Kit (Qiagen) was used in a 384 well format. From each sample 12 ng template cDNA was used in a total volume of ten microliters. The reactions were carried out in triplicates in a LightCycler 480 (Roche). The endogenous controls ACTB, B2M and TUBA3C were used for normalization.

### Viability assay

MDA-MB-231 cells were seeded in 96 well microplates at 300 cells per well. After 24 h, 15 μl of lentiviral supernatant (approx. 1000 units) in culture medium containing 8 μg/ml polybrene was added to the cells to achieve a MOI > 1. Twenty four hours later the viral medium was aspirated and replaced by culture medium containing 0.5 μg/ml puromycin or culture medium without puromycin, respectively. 72 h post-infection puromycin selected and non-selected cells were assayed by resazurine assay in triplicate measurements (t_zero_). The fluorescence intensity ratio from puromycin selected cells divided by the intensity from unselected cells was used as quality control for efficacy of lentiviral infection. Another triplicate was allowed to proliferate in fresh puromycin culturing medium for another five days before resazurine measurement (t_end_). The fluorescence intensity ratio [t_end_/t_zero_] served as a relative measure for the anti-proliferative effect of tested shRNA constructs. All values were normalized to a non-silencing control (NSC) as well as an empty-pGIPZ vector control. For the viability assays at sixteen days post infection total cells were transferred from a well of a 96 well plate to that of a six well plate at six days post infection.

### Caspase activation assay

MDA-MB-231 cells were seeded in 96 microwell plates at 300 cells per well. After 24 h, 15 μl of lentiviral supernatant (MOI >1) in culture medium containing 8 μg/ml polybrene was added to the cells. Twenty four hours later the viral medium was aspirated and replaced by standard cell culture medium. At three and six days post-infection a Caspase-Glo 3/7 Assay (Promega) was performed. Luminescence was detected in a Fluorostar plate reader (Perkin Elmer) after one hour of incubation at room temperature.

## Authors' contributions

MB designed microarray layouts and performed microarray experiments as well as validation assays, led the data analysis and drafted the manuscript. JF produced the lentiviral pools, assisted with performance of candidate validation assays and provided comments on the manuscript. AMG performed the bioinformatic analyses. YH, ID, DC and JDH provided advice on experimental design and provided comments on and revisions to the manuscript. DC and JDH provided the original concept for the study and supervised the study. All authors read and approved the final manuscript.

## Supplementary Material

Additional file 1 Results from negative selection screen, Shown are the clone IDs as well as stem sequences and target genes from all 305 shRNA expression constructs included in the screen. Click here for file

Additional file 2 Results from negative selection screen, From the 278 constructs analyzable by the described conditions, (t_end_/t_zero_) log_2 _ratios are summarized from individual tiling probes as well as mean log_2 _ratios and *p*-values for each shRNA expression construct. Click here for file

Additional file 3 Description: Array data deposition information, Shown are ArrayExpress accession numbers. Click here for file
